# Antiscalants Used in Seawater Desalination: Biodegradability and Effects on Microbial Diversity

**DOI:** 10.3390/microorganisms10081580

**Published:** 2022-08-05

**Authors:** Ashraf Al-Ashhab, Amer Sweity, Luna Al-Hadidi, Moshe Herzberg, Zeev Ronen

**Affiliations:** 1 The Dead Sea and Arava Science Center, Masada 86190, Israel; 2 Eilat Campus, Ben-Gurion University of the Negev, Eilat 8855630, Israel; 3 National Agriculture Research Center (NARC), Baqa’a 19381, Jordan; 4 Zuckerberg Institute for Water Research, Jacob Blaustein Institutes for Desert Research, Ben-Gurion University of the Negev, Sede Boker Campus, Midreshet Ben-Gurion 8499000, Israel

**Keywords:** biodegradation, antiscalants, desalination, microbial diversity

## Abstract

Antiscalants are organic polymers widely used for scale inhibition in seawater desalination. While they are susceptible to biodegradation, they provide nutrients for bacterial cell growth and energy for the microbes that assimilate and degrade them. This paper shows the biodegradability of three commercial antiscalants (polyacrylate—CA, polyphosphonate—PP, and carboxylated dendrimers—DN) applied in seawater reverse osmosis desalination (SWRO) as well as analyzing the antiscalant’s effects on microbial diversity using microbial cultures grown in seawater, under semi-continuous batch conditions. Nutritional uptake and contribution of the antiscalants to microbial growth were investigated by measuring DOC, TDN, NO_3_^−^, NO_2_^−^, PO_4_^−^, NH_4_^+^, and TP of the filtered samples of the incubated batch, twice a month, for twelve months. The microbial community was estimated by 16S rRNA sequencing. The main changes in the microbial communities were determined by the incubation period. However, bacterial orders of the antiscalant treatments differed significantly from the control treatment, namely *Planctomycetales*, *Clostridiales*, *Sphingobacteriales*, *Rhodobacterales*, and *Flavobacteriales*, and other unclassified bacterial orders, which were found in various relative abundances dependent on incubation times. The results showed the PP antiscalant to be the least biodegradable and to have the least effect on the bacterial community composition compared to the control. This result emphasizes the need to reassess the suitability criteria of antiscalants, and to further monitor their long-term environmental effects.

## 1. Introduction

Due to the multiple uses and increasing industrial applications of antiscalants [1,2], especially in desalination facilities [3,4], tons of thousands of these chemicals are discharged into the environment every year, causing detectable environmental impacts. Antiscalants may negatively impact marine environments and affect fish life, coral reefs, sea-grass meadows, zooplankton, and microbial communities [5,6,7,8]. At desalination plants and facilities, restrictions implemented on brine discharge have pushed the scale-inhibitor industry to develop biodegradable and environment-friendly antiscalants [8,9,10]. Researchers consider antiscalants with 60% degradation capability within 28 days as a biodegradable material [11]. Although polyphosphonate-based antiscalants are considered stable, some researchers have reported that they are biodegradable by some microorganisms, including some halophilic bacteria, at different rates [12]. Polyacrylate-based antiscalants are also susceptible to biodegradation in marine environments: it was found that 52% of polyacrylic acids degraded after 35 days of disposal [13]. Nonetheless, biodegradable antiscalant discharge may pose many environmental risks and concerns [14,15,16,17].

For instance, polyphosphate-based antiscalants can be readily hydrolyzed to orthophosphate by cleaving the O–P bonds, which are considered a significant nutrient source for heterotrophic microorganisms and phytoplankton [18]. In contrast, polyphosphonate antiscalants possess resistant C–P bonds which lower degradation rates and lengthen residence times in coastal waters [19,20]. Biodegradation of antiscalants, such as amniotic [21], may release amino-methyl-phosphonic acid as a metabolite, which is considered a major issue in environmental protection [22,23,24]. On the other hand, antiscalants may also influence natural mineral processes in the marine environment; for instance, phosphate-based antiscalant degradation was suggested to induce an oligotrophic marine environment, affecting the aquatic microbial community composition and causing cyanobacteria blooms [25,26,27]. Researchers showed that increased marine water eutrophication, caused by desalination plant discharge, significantly increased nitrogen and phosphorus concentrations, affecting coral reefs, fish, zooplankton, and marine microbes [7,8,17,28,29].

Antiscalants can serve as nutrients for microbial growth, especially in oligotrophic environments such as in seawater with low concentrations of dissolved organic carbon, phosphorus, and nitrogen [12,30,31]. The growth of marine microorganisms is limited by nutrient availability, especially fixed inorganic nitrogen and phosphorus. Polyacrylate (CA) and carboxylated dendrimer (DN) based antiscalants are commonly dosed to the seawater feed during the desalination processes at very low concentrations [32]. Hence, under oligotrophic conditions, they may significantly increase the dissolved organic carbon concentration, which consequently enhances membrane biofouling. While previous studies used plate culturing microbial-growth-based isolation techniques for microbial characterization, our goal was to investigate how the main types of antiscalants affect bacterial diversity and bacterial community structure with relation to membrane biofouling and the consequent changes in the discharged microorganisms. We focused on commercial antiscalants based on carboxylate (DN)-, acrylate (CA)- or phosphonate (PP)-based chemistry ([1,2] and Supplementary Materials, Figure S1), while previous studies used monomers and polymers of known anti-scaling inhibitors [12]. The objective of this study was to investigate the biodegradation potential of these three commercial antiscalants, commonly used in seawater desalination plants, and to explore the effects of these compounds on the microbial community structure under marine aquatic conditions.

## 2. Materials and Methods

### 2.1. Antiscalants

Three commercial antiscalants (chemical structure of the main active content is shown in Supplementary Materials, Figure S1): (i) polyphosphonate-based (PP) (Genesys Int’l, Cheshire, UK), (ii) polyacrylate-based (CA) (Genesys Int’l, Cheshire, UK), and (iii) carboxylated dendrimeric-based (DN) Spectraguard (Vista, CA, USA) were obtained in a liquid form.

### 2.2. Enrichment Culture

Seawater samples were obtained from the Palmachim desalination plant. In order to provide the biofouling potential of the antiscalants under realistic conditions, the samples being tested underwent flocculation, coagulation, and sand filtration at the plant [33]. These pretreated seawater samples used for the enrichment cultures (500 mL) were supplemented with different antiscalants, at concentrations of 100 mg/L in sterile 1 L Erlenmeyer flasks. The relatively high antiscalants concentration of 100 mg/L applied was to support an enhanced microbial activity and growth and to conduct the study within a reasonable research time frame of one year. Each of the three antiscalant enrichment experiments was replicated six times to provide sufficient sampling volume and replicates (3 types of antiscalants × 6 of 500 mL replicate experiments). In addition, an enrichment culture without antiscalant was served as a control (seawater only) to provide the impact of antiscalants on the structure of the developed microbial communities. The flasks used for the enrichment tests were placed in a 12:12 h, light–dark, cycle at 25 °C with continuous agitation of 250 rpm for one year.

During the incubation period, 450 mL samples were taken (after one week, two weeks, four weeks, three months, six months, and a year of incubation) and then filtered through sterile 0.22 µm Supor filter papers (47 mm, Pall Gelman, Ann Arbor, MI, USA); the filtrate was used for chemical analysis and the filter papers were stored in −80 °C for later analysis of microbial community. After each period of sampling, new 450 mL of pretreated seawater samples were added directly to the remaining 50 mL of the old, incubated experiment. In this way, an enrichment ratio of 1:9 was achieved at every sampling time point (one week, two weeks, four weeks, three months, six months, and a year of incubation).

### 2.3. Chemical Analysis

The dissolved organic carbon, total dissolved nitrogen, total phosphorus, ammonium, and volatile suspended solids of the culture media were measured to assess the degradation kinetics of the antiscalants [34].

### 2.4. DNA Extraction, Library Preparation, and Sequencing

Following filtration, the filter papers were cut into two; one half was used for DNA extraction, and the other half was placed in −80 °C. The DNA was extracted using MoBio PowerSoil™ DNA extraction kit (MoBio, Carlsbad, CA, USA) following the manufacturer’s protocol.

*Following DNA extraction, the V3 region of the 16S rRNA gene was amplified using universal bacteria Eub-341F (5′-CCTACGGGAGGCAGCAG-′3) [35] and Eub-519R (5′-GWATTACCGCGGCKGCTG-′3) [36] PCR primer. PCR reactions were performed in triplicate as previously described [37]. Following PCR reaction, replicate samples were pooled together and cleaned using PCR cleaning kit (Sigma, Jerusalem, Israel) and the product was sent to RTSF Genomics Core (Michigan State University, Chicago, IL, USA) for 454 pyrosequencing.*

### 2.5. 16S rRNA Sequence Analysis

Screening for high-quality sequences, alignment, chimera detection, and PCR noise removal was performed with the Mothur software package [38]. All sequences were aligned based on the SILVA bacteria reference alignment database [39], and sequences with minimum average quality scores of 25 were retained before sequence alignment. After alignment, sequences were screened for possible chimeric sequences using the chimera.uchime algorithm implemented in Mothur. PCR noise was removed by Single Linkage Pre-clustering, as described previously [40]. Sequences were then classified using the classify.seqs command in Mothur. After classification, sequences identified as Mitochondria, Chloroplast, and Unknown_Bacteria were removed, and an operational taxonomic unit (OTU) table based on 97% similarity was generated. Diversity estimates, including the Shannon–Wiener diversity index [41] and the species richness estimator Chao1 [42], were calculated, and the bacterial taxonomic group was allocated to the family level. Bacterial relative abundance was set as the number of sequences affiliated with that taxonomic level divided by the number of sequences per sample.

### 2.6. Statistical Analysis

All data processing, figure generation, and statistical tests were performed in R and made available on GitHub respiratory (https://github.com/ashrafashhab/Anitscalent_Biodegradation.git accessed on 9 August 2021). A Permutational Multivariate Analysis of Variance (adonis) statistical test measured the statistical differences of the various antiscalants and incubation times. PCA ordination was used to visualize distinct bacterial community differences and corrected Anova was performed on the normalized chemical analysis. RDA analysis was performed to investigate the chemical composition of the microbial communities. In parallel, we generated a heat map with a bacterial order of >5% of total bacterial abundance to explore the microbial communities in relation to different antiscalants and incubation time.

## 3. Results and Discussion

This study investigated the biodegradability of antiscalants used in seawater desalination and, for the first time, their effect on the microbial communities abundance and diversity in a continuous pot lab microbial enrichment experiment design. Three major types of commercially available antiscalants were tested for their biodegradation and the associated microbial communities that developed over a 12-month period.

### 3.1. Antiscalant Biodegradability

**Figure 1:**
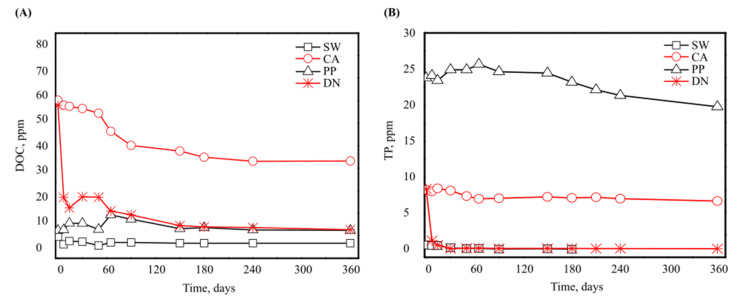
DOC (**A**) and TP (**B**) concentrations of the incubated seawater in the presence and absence of 100 mg/L polyacrylic acid- (CA), polyphosphonate- (PP), and carboxylated dendrimer- (DN) based antiscalants over one year of incubation.

**Figure 2:**
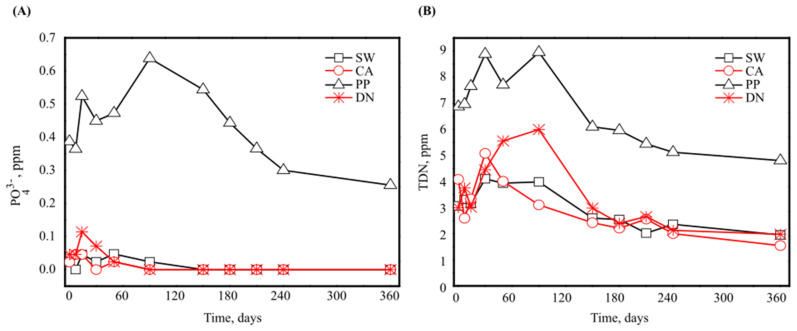
Phosphate (PO_4_^3−^) (**A**) and TDN (**B**) concentration of the incubated seawater in the presence and absence of 100 mg/L polyacrylic acid- (CA), polyphosphonate- (PP), and carboxylated dendrimeric- (DN) based antiscalants.

The biodegradability of antiscalants was measured through a set of chemical analyses: Dissolved Organic Carbon analysis (DOC), Total Phosphorus (TP) analysis, Total Nitrogen (TDN), ammonium (as N), and nitrite (N-NO^3−^) (Figure 1 and Figure 2 and Supplementary Figure S2). Figure 1 shows the average values for DOC (Figure 1A) and TP (Figure 1B) for the entire enrichment period in the culture enriched medium with DN, PP, CA antiscalants, and control (SW). Each antiscalant has a unique nutrient composition. For instance, CA is richer in carbon than PP antiscalant, which is richer in TP. As expected, carbon-rich CA and DN showed the highest DOC initial concentration (Figure 1A), compared to PP, which had the highest initial TP concentration (Figure 1B).

Figure 1 also indicates the biodegradability of different antiscalants over the one-year incubation period; the rapid biodegradability of DN during the first few days of incubation is evident (Figure 1A). For instance, assuming first order degradation kinetics, the DOC declined at rates of 1.05 × 10^−1^ and 2.7 × 10^−3^ day^−1^ for DN and CA antiscalants, respectively (Supplementary Materials, Figure S3). In the DN supplemented media, DOC concentration decreased rapidly from 60 to 11 ppm during the first days of incubation (Figure 1A), and TP concentration decreased from 8 to almost 1.3 ppm (Figure 1B). These concentrations continued to gradually decline to nearly 10 and 0.2 ppm for DOC and TP, respectively, throughout the incubation period. Second to DN, CA antiscalant degraded by 40% during the first 120 days (Figure 1A); DOC concentration dropped from 60 ppm to 35 ppm and remained constant afterward. The degradation is also evident in the decrease of TP, reaching 8.2 ± 1.1 ppm at the end of the experiment (Figure 1B). In contrast, PP antiscalant exhibited the lowest biodegradability; its TP concentration declined slowly to reach 22 ± 3.3 ppm at the end of the incubation period.

Interestingly, when measuring phosphate as PO_4_^3−^ and total dissolved nitrogen (TDN) concentrations (Figure 2), a considerable increase was noticed in PP and DN during the first 90 days of incubation (for PP: the initial PO_4_^3−^ concentration increased from 0.4 to 0.65 ppm and the initial TDN increased from 7 to 9 ppm; for DN: the initial TDN concentration increased from 3 to 6 ppm). This initial increase in nitrogen concentration was also shown when measuring ammonium and nitrate concentrations (Supplementary Figure S2). After 90 days of incubation, PO_4_^3−^ began a gradual decline, eventually reaching 0.28 ppm and undetected levels for PP and DN antiscalants, respectively (Figure 2A). The gradual decline in TDN was observed to reach 5 and 2 ppm for PP and DN antiscalants, respectively (Figure 2B). This fluctuation and initial increase in PO_4_^3−^ and TDN for PP and DN antiscalants could be attributed to these materials’ higher P and N composition. The initial increase may be related to a release of these nutrients to the medium by specific extracellular degradation of the dissolved polymers. The later decline could be related to the specifics of biodegradation and microbial uptake [18,43].

Likely, the rapid degradation of DN antiscalant (Figure 1) is carried out through microbial processes. It has been suggested that different antiscalants serve as carbon and energy sources for seawater bacteria [30]. These macromolecules were shown to be assimilated and mineralized as carbon and energy sources for further microbial growth [42]. Hence, generating energy by activating various enzymes and cleaving the polymer bonds, producing oligomers, dimers, and monomers [44]. It was reported that denitrification capacity increases with an increase in DOC content [45]. DOC determines the ability of microorganisms to conduct their continuous functions. For example, bacteria play a pivotal role in ecosystems; they are responsible for processes including nitrification and mineralization of organic matter. Larson et al. (1997) showed that the mixed microbial community from activated sludge used acrylic acid antiscalant as a carbon and energy source [13].

Similarly, DN and CA antiscalants showed the highest concentrations of DOC. Nonetheless, the rate of DN biodegradation was the highest. The differentiation between DN and CA biodegradation rates may be attributed to an easier degradation and digestion of DN polymers, since they may contain more readily utilized phosphorus (a faster decline in P concentration as shown in Figure 1B) and a relatively high content of biodegradable C (Figure 1A) [46].

As expected, PP antiscalant presented the least biodegradability (Figure 1A,B) due to the PP antiscalant’s initial low carbon contribution and C–P bond stability [47]. In this context, P is an essential nutrient causing eutrophication in marine environments [48,49] as primarily microorganisms needed it for biosynthesis of nucleic acids, phospholipids (cell membrane), and adenosine triphosphate (ATP) for energy [50]. Although biodegradability of PP is low, its contribution of nitrogen and phosphorus is substantial in oligotrophic seawater conditions, as it serves as a labile nutrient source of nitrogen and phosphorus even if it is slowly degraded and utilized [51].

### 3.2. Microbial Community Analysis

According to the previously measured chemical composition, the different antiscalants degrade at different rates; the high bioavailability of P shown for the dendrimeric antiscalant could explain the rapid C consumption (DN, Figure 1A,B). The differences in the chemical composition and the associated degradation rates of the applied antiscalants are expected to determine the microbial communities and diversity patterns, which may affect biofouling scenarios or eutrophication in marine hotspots during brine discharge. Therefore, we investigated the bacterial diversity and community composition in the presence of different antiscalants after 30, 60, and 90 days and at the end of the one-year incubation period. The bacterial diversity was measured by a set of indices indicating bacterial richness (Choa1) [42], and bacterial community diversity index (Shannon–Wiener) [41], while bacterial community composition was measured by taxonomic assignment of the obtained OTUs.

#### 3.2.1. Bacterial Diversity

418,738 high-quality bacterial 16S rRNA-encoding gene sequences were obtained with an average of 27,915 ± 18,805 (75.6 ± 6.5% of row sequences) sequences per data set (Supplementary Table S1). First, to ensure the obtained sequences represent the actual bacterial diversity in our samples, we estimated sample coverage (ESC) using Good’s coverage index (Supplementary Table S1). Good’s coverage index showed a high ESC and ranged from 95.4–98.9%, meaning only 1.1–4.6% of the obtained sequences appeared once. The high ESC indicates that the obtained sequences reflect the actual bacterial diversity in the samples [52]. As good coverage was obtained throughout the samples, bacterial species richness was measured by the Chao1 index (Supplementary Table S1); the results showed an increased species richness when antiscalants were added compared to the control (SW). At 30 days of incubation, DN antiscalant showed to have the highest species richness (1065) when compared to PP (936), CA (856), and SW (659). The high species richness in the DN antiscalant can be attributed to its high biodegradability rate. Figure 1 and Figure 2 and Supplementary Figures S2 and S3 show that the DN antiscalant is the most biodegradable; immediately after incubation (between days 0 and day 60), it releases nutrients for bacterial growth and the highest increase in OTUs occurs after 30 days of incubation. On the other hand, after 60 days of incubation, PP and CA antiscalants showed the highest species richness (2413 and 1939) compared to DN (1217) and SW (957). The observed increase in OTUs at 60 days of incubation is believed to be related to an increase in both TP and DOC, where TP is the limiting factor [53,54]. Previous research indicated that bacterial cell volumes, protein production rates, and abundances increased when lake water was enriched with phosphate alone; however a higher increase in abundances was observed when glucose was added [55]. In our study, the measured TP concentration was also the highest for PP, followed by CA antiscalant (Figure 1B), while DOC concentration was higher for CA compared to DN (Figure 1A). These nutritional conditions may explain the increase in species richness for different antiscalants in the order of PP > CA > DN > SW, suggesting that TP determines increased species richness more than TN, a finding that was also documented previously in Mediterranean seawater [56]. Interestingly, after 90 days, as well as after 1 year of incubation, Choa I richness declined gradually for each antiscalant; yet after one year, species richness was higher compared to 30 days of incubation except for DN, which was higher at day 30. After 1 year, DN’s species richness was similar to the control (SW) (Supplementary Table S1). In relation to nutrient concentrations, Figure 1 and Figure 2 indicate that DN completely degraded at the early stages of incubation. In contrast, after 90 days, PP antiscalant showed a gradual decrease in DOC and TP concentration continuing for the entire period of one year experiment (Figure 1). Similarly, CA showed a steady decline in TP concentration (Figure 1B).

While bacterial diversity measured by the Choa I index represents species richness, the Shannon–Wiener index accounts for both abundance and evenness. The differences between those two indices can give good insights into environmental perturbation (i.e., a sudden increase in nutrients) and different bacterial adaptation strategies k/r-strategies [57]. For instance, the release of nutrients accompanied by antiscalant degradation can enhance species richness through bacteria exhibiting r-strategy, reducing community evenness, thus reducing the Shannon–Wiener index (i.e., CA at 30 days of incubation, Supplementary Table S1). On the other hand, an increased Shannon–Wiener index indicates a more stable microbial community (notice high Shannon–Wiener index for control). Interestingly, after one year of incubation, microbial consortia supplemented with PP antiscalant had a slightly higher Shannon–Wiener index than the CA antiscalant, indicating that the PP antiscalant supports a more stable consortia and has minimal effect on the microbial community (Supplementary Table S1).

#### 3.2.2. Effect of Different Types of Antiscalants and Incubation Time on the Bacterial Community Composition

**Figure 3:**
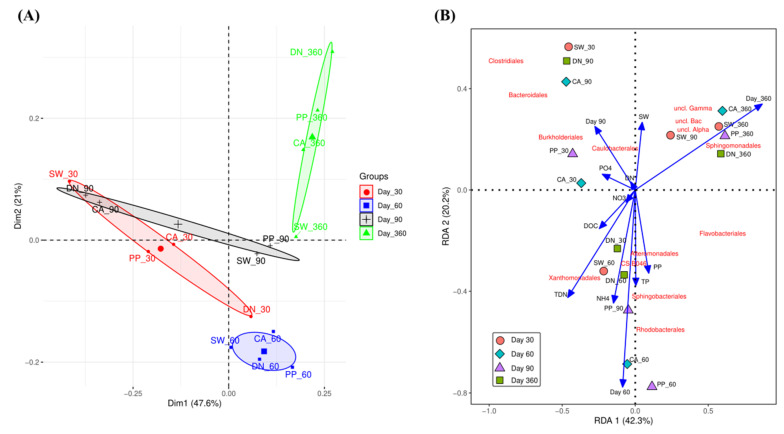
PCA (**A**) and RDA (**B**) ordination for different antiscalants and for different incubation times.

To investigate the effect of (i) types of antiscalant and (ii) incubation time on the microbial communities, we aggregated all OTUs to order level. Statistical tests were performed using Permutational Multivariate Analysis of Variance Using Bray–Curtis Distance Matrices (adonis) (Supplementary Table S2). The results show incubation time (*p*-value = 0.001) but not types of antiscalant (*p*-value = 0.754) to significantly affect the bacterial community compositions. The significant effect of incubation time was also clear when PCA ordination was plotted, the PCA plot in Figure 3 shows bacterial community association (similarity or dissimilarity) to cluster mainly according to the different incubation times (Figure 3A). Other consortia treated with different types of antiscalants did not cluster separately, indicating no differences in the microbial communities based on the anticalants added to their media. However, separate clusters were observed for different incubation periods. At day 60 (blue ellipse) and after 1 year of incubation (day 360, green ellipse), both bacterial communities showed clusters clearly separated from each other. Interestingly, the control treatment at those two time points (SW-60 and SW-360) also clustered according to incubation period. Figure 3 also shows that the most significant differences between types of antiscalants were present at day 30 and day 60. On day 30 (red ellipse), SW-30 was significantly distinct compared to CA-30 and PP-30, while DN-30 showed the highest dissimilarity compared to the control (SW-30). At day 60, both CA-60 and DN-60 clustered together and were separated from PP-90 and SW-90, which also clustered together. Notably, CA-60, DN-60, and SW-30 were close to each other, indicating similar bacterial communities (Figure 3A).

Interestingly, Figure 3A shows that experimental incubation setup, without the addition of any antiscalant, significantly affected the microbial communities. Looking at the control treatment (SW), we see the biggest difference of our enriched cultures occurred at the x-axis between day 30 and day 60 and notably, the x-axis explains 47.6% of the total bacterial variance. At the same time, the control SW-60, SW-90, and SW-360 were closer to each other and showed separation by the y-axis, which explains 21% of the total microbial variance. To explain these changes along with the set of different measured chemical compositions (Figure 1, Figure 2 and Figure S1), we performed a Redundancy Analysis (RDA, Supplementary Table S3) and generated a biplot for the different samples along with the measured chemical composition and significantly abundant bacterial Order_classification (Figure 3B). While the RDA analysis of corrected significance ANOVA did not show any significant effect for measured chemical data (Supplementary Table S3), the bioplot (Figure 3B) shows some interesting results. We see most measured chemical analyses correlated (length and direction of the blue arrows) with samples taken at day 60 except for DN-30 and PP-90, which also clustered with samples taken at day 60 and correlated with chemical analyses (recall degradation pattern for DN and PP antiscalant with a peak at day 90 for PP and at day 30 for DN, which also correlates to a higher increase in TDN and NH_4_^+^ concentrations as shown in Figure 2B, Figure 3B and Figure S1). Figure 3B also shows that the microbial community compositions after one year do not correlate with any of the chemical compositions. The disparity is mainly due to the presence of *Sphingomonadales*, *Uncultured_Bacteria*, *Uncultured_Gammaproteobacteria*, and *Uncultured_Alphaproteobacteria* orders. We cannot draw a decisive conclusion about the characterized uncultured bacterial kingdom, phyla, class, or orders, since these bacterial groups can contain a wide range of distinctive bacterial activities. Yet, in the following sections, we will comment on the common characteristics shared among these bacterial orders in light of our experiment. For instance, the elucidated Sphingomonadales are heterotrophic, consisting of some species that are phototrophic with unique exopolysaccharide producing capabilities. For samples taken at day 30 (except for DN-30), the biomass seemed to cluster together along with DN-90 and CA-90. Notably, high degradability detected for DN followed by CA-supplemented cultures may explain the clustering after 90 days of incubation as nutrient consumption. Hence, the dominant microbial communities at day 60 were *Xanthomonadales*, *Alteromonadales*, *Flavobacteriales*, *Caulobacteraceae*, and *Burkholderiales*. These bacterial orders were found to be important in nitrate reduction (Xanthomonadales); widely available in sea waters (*Alteromonadales*); while some possess chemoorganotrophic properties (*Flavobacteriales*, *Caulobacteraceae*, and *Burkholderiales*). Notably, *Sphingomonadales* and *Flavobacteriales* are both known to have heterotrophic and phototrophic properties with a high ability to form a biofilm. This may explain their higher presence after one year of incubation.

#### 3.2.3. Bacterial Community Composition

##### Bacterial Phylum Composition

To identify the main bacterial community composition in the different cultures supplemented with different types of antiscalants, relative abundance was compared for all found bacteria (Supplementary Materials, Figure S4—different shades of the same color represent different bacterial order under the same phylum classification). The results show four major bacterial phyla being abundant across all samples with temporal variations: *Proteobacteria* (48.7 ± 18.1%), *Bacteroidetes* (23.4 ± 7.7%), *Firmicutes* (15.1 ± 18.5%), and *Planctomycetes* (6.4 ± 11.6%). Figure S2 also shows that different antiscalants significantly affect the microbial community composition compared to the control experiment: after 30 days of incubation, a higher abundance of *Proteobacteria* (ranging from 43.7 to 56.0%) and lower abundance of *Firmicutes* (ranging from 6.4 to 32.0%) were observed, compared to control seawater bacterial community (16.8% and 53.6% for *Proteobacteria* and *Firmicutes*; respectively). However, on day 60, all samples had similar *Proteobacteria* abundance (65.8 ± 1.4%). Figure S2 also shows a significant change of bacterial communities in the control treatment (SW), just after 30 days of incubation, *Firmicutes* abundances significantly decreased and reached a stable community composition after 60 days of incubation while after 30 days of incubation (the 1st sampling point), *Firmicutes* relative abundances were significantly lower when different antiscalants were added. Interestingly, when seawater was supplemented with different antiscalants, a remarkable shift in the microbial communities was also noticed after 90 and 360 days of incubation. After 90 days of incubation, *Firmicutes* abundances significantly increased in both CA (43.6%) and DN treatments (46.4%) but not in PP treatment (4.1%), indicating that different antiscalants can alter the microbial community composition differently (Figure 3A). After 360 days of incubation, each antiscalant had a significant effect on the microbial communities, resulting in unique community compositions. A significant increase in planctomycetes was observed in DN, CA, and PP treatments (45.4, 19.2, and 13.4%) compared to control SW (2.6%).

##### Bacterial Order Compositions

**Figure 4:**
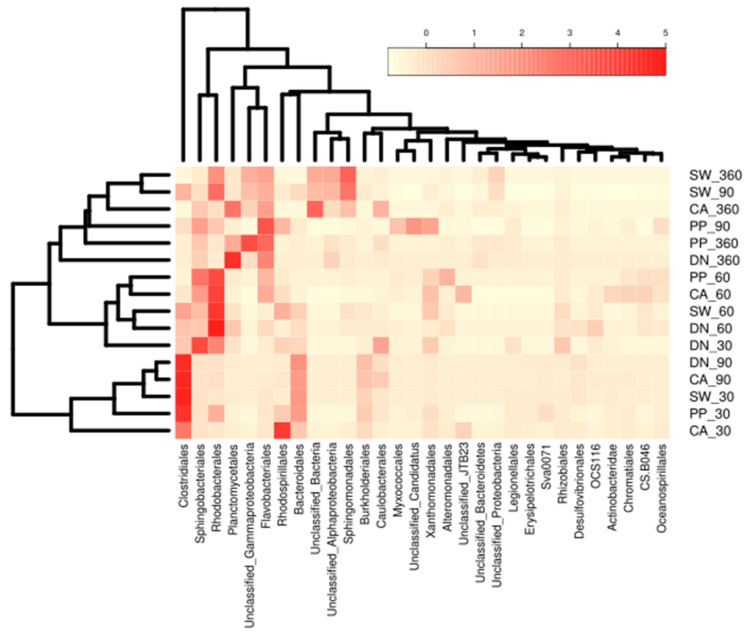
Heat map showing the bacterial orders (>5% of total abundance) at different time points and treatments.

To investigate the variation between different treatments, we generated a heat map (Figure 4, Supplementary Table S4) for bacterial order whose total abundance is higher than 5%. We concluded that ca. 30 bacterial orders form the core microbial communities, while some of the bacterial orders are distinctive for different time points as well as for different antiscalant treatments. Planctomycetales were mostly abundant after one year of incubation; their relative abundances in the antiscalants followed DN > CA > PP > SW with 45.4, 19.2, 13.4, and 2.6% respectively. Planctomycetales are chemolithotrophic bacteria are known for their ability to form biofilms [58,59]. They have a relatively slow growth cycle; the doubling time of some Planctomycetes can reach 30 days [60]. Their high capacity to break down extremely complex carbohydrates [61,62,63] may explain their highest abundance after one year of incubation time. Similarly, Flavobacteriales were the second most abundant bacteria after one year of incubation, following PP > DN > CA > SW at relative abundances of 26.4, 25.4, 12.8, and 11.9%, respectively. A recent study indicated their role in intermediate oil and complex hydrocarbon degradation [64], which also may explain their high abundance after one year of incubation, along with Planctomycetales. Yet, their highest abundances were in PP antiscalants, and their relative abundance in PP gradually increased after incubation, following the pattern of 30 < 60 < 90 < 360 days with relative abundances of 2.3, 10.0, 19.4, and 26.4% respectively. The *Clostridiales* order belonging to the *Firmicutes* phylum were mostly abundant after 30 days of incubation except for DN_30, which was mainly dominated by the Sphingobacterilaes belonging to the Bacteroides phylum. The *Clostridiales* order found here are a very diverse order of obligate anaerobes and are important in bioremediation processes [65,66,67]. *Clostridiales* were also found in seawater desalination processes as well as in biofilms being developed on the expense of antiscalants [68,69,70]. Bacteria from the *Sphingobacteriales* order were already shown to induce EPS production by biofilms developed at the expense of different types of antiscalants during desalination process [71]. Following 60 days of incubation (when most degradation of antiscalants occurs, Figure 1 and Figure 2), we observe a remarkable abundance of *Rhodobacterales* order belonging to *Proteobacteria*, forming 28.5% of total bacterial communities. *Rhodobacterales* was previously reported in the brines of desalination plants having a similar magnitude of the total bacterial communities of ~43% when phosphate-based antiscalants were added [27]. It was also reported that *Rhodobacterales* contain phosphonate ABC transporter genes and C–P lyases that cleave the C–P bond of phosphonates [43,72]. This relative enrichment in *Rhodobacterales* was reported to enhance the P bioavailability to the entire microbial community and provide nutrients to groups such as *Flavobacteriales*, whose relative abundance greatly increased when PP antiscalant was added [68]. Interestingly, for the PP antiscalant, we see a similar result in this study—an increase in the abundance of *Flavobacteriales* after 90 days of incubation; the relative abundance of *Flavobacteriales* at PP_90 was 19.4% compared to 2.3% and 10.0% for PP_30 and PP_60, respectively. Notably, a further increase in the *Flavobacteriales* abundance to 26.4% was observed after 360 days of incubation with PP antiscalant. Similarly, the abundance of the *Flavobacteriales* order increased also for the DN antiscalant after 360 days of incubation, reaching 25%, while the CA_360 was similar to the SW_360 control (12.8 and 11.9%, respectively).

## 4. Conclusions

Antiscalants can serve as alternative sources of nutrients which support microbial growth in oligotrophic environments, where the dissolved organic carbon, phosphorus, and nitrogen concentration are limited. Our results indicate that antiscalants affect microbial diversity and bacterial community compositions. Both incubation time and different types of antiscalant—to a less extent—showed to significantly affect the microbial communities. The bacterial orders elucidated in this study were found to be highly relevant to the antiscalants’ degradation: Chemoorganotrophic biodegrading bacteria and other bacterial orders that specialize in the degradation of C–P bonds were all found to be significantly abundant in a certain antiscalant over the other. The biodegradability of antiscalants along with the changes in the microbial communities were the highest for DN and least for PP (DN > CA > PP). Interestingly, PP antiscalants, the least biodegradable, were most similar to control samples (Figure S2). Whether application of PP antiscalants is most favorable due to the least alteration of microbial communities, or DN antiscalants due to its high biodegradability, further investigation and long-term monitoring will be required, including the potential effect of different antiscalants on other marine organisms such as seagrass meadows and reef-building coral species.

## Data Availability

Sequences used in this study were submitted to the MG-RAST (https://www.mg-rast.org/linkin.cgi?project=mgp18790, accessed on 9 August 2021) under accession numbers (4706723.3-4706726.3 on 6 July 2016). All statistical analyses and figure generation code was performed in R has been made publicly available and can be found in GitHub by clicking on this link (https://github.com/ashrafashhab/Anitscalent_Biodegradation.git, accessed on 9 August 2021).

## Supplementary Materials

The following supporting information can be downloaded at: https://www.mdpi.com/article/10.3390/microorganisms10081580/s1, Figure S1: N-ammonium (A) and N-NO^3−^ (B) concentration of the incubated seawater in the presence and absence of 100 mg/L polyacrylic acid (CA), polyphosphonate (PP), and carboxylated dendrimeric (DN) based antiscalants; Figure S2: Phyla relative abundance in each treatment following different incubation periods. Shades of the same color represent the different bacterial order in each phylum; Figure S3: DOC degradation kinetics of 100 mg/L polyacrylic acid- (CA) and carboxylated den-drimeric- (DN) based antiscalants. Figure S4: Phyla relative abundance in each treatment following different incubation periods. Shades of the same color represent the different bacterial order in each phylum. Table S1: A number of high-quality sequences, Good’s coverage, Chao1, and Shannon-Wiener index for all studied treatment and different time points; Table S2: Adonis significance based on Bray-Curtis distance matrix; Table S3: ANOVA analysis of Adjusted R2 for RDA analysis showing different variables and chemical analysis significance and percent of the variation.
