# Supplementary material for: Antiscalants Used in Seawater Desalination: Biodegradability and Effects on Microbial Diversity
Source: Microorganisms. 2022 Aug 5;10(8):1580. doi: 10.3390/microorganisms10081580 (PMC9414044; doi:10.3390/microorganisms10081580)
Supplement: Supplementary file 1 [file microorganisms-10-01580-s001.zip › microorganisms-1827236-supplementary.pdf]

microorganisms

Supplementary Materials

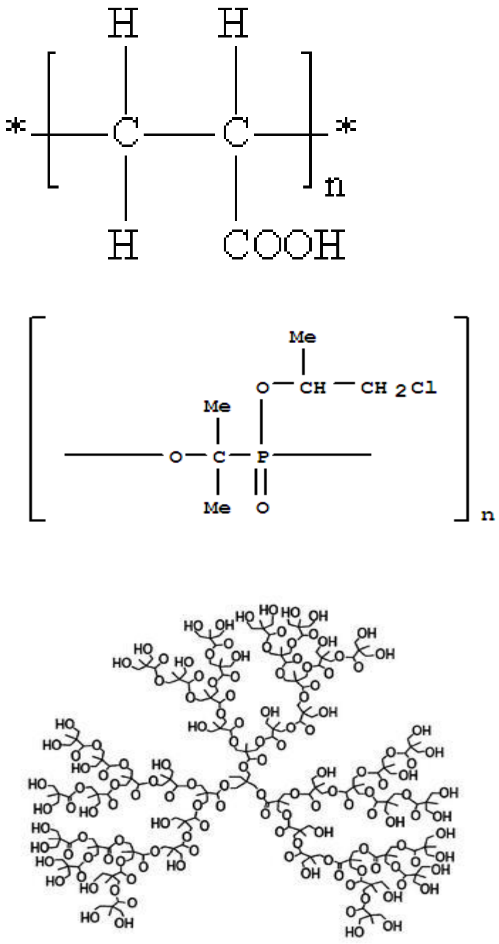

Figure S1. The active antiscaling polymers content in the commercial antiscalants used in this study, from top to bottom: Polyacrylic-acid; Polyphosphonate; and Carboxylated dendrimeric polymer.

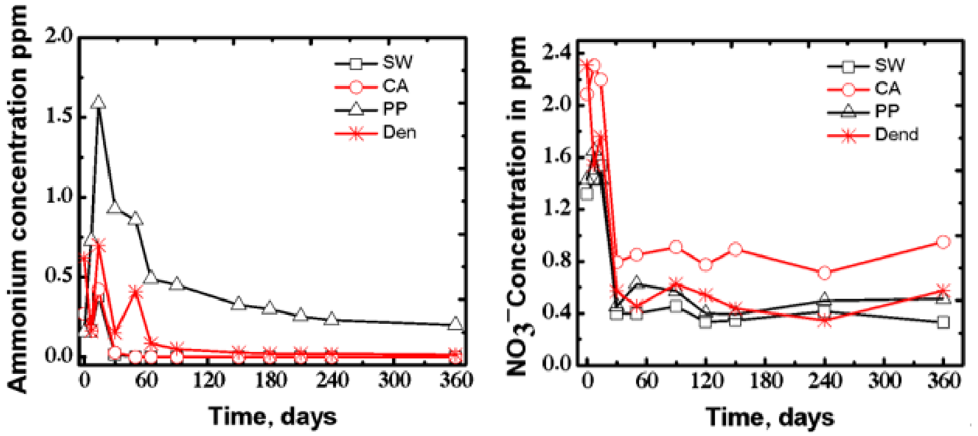

Figure S2. N-ammonium (right) and N-NO^{3-} (left) concentration of the incubated seawater in the presence and absence of 100 mg/L polyacrylic acid- (CA), polyphosphonate- (PP), and carboxylated dendrimeric- (Den) based antiscalants.

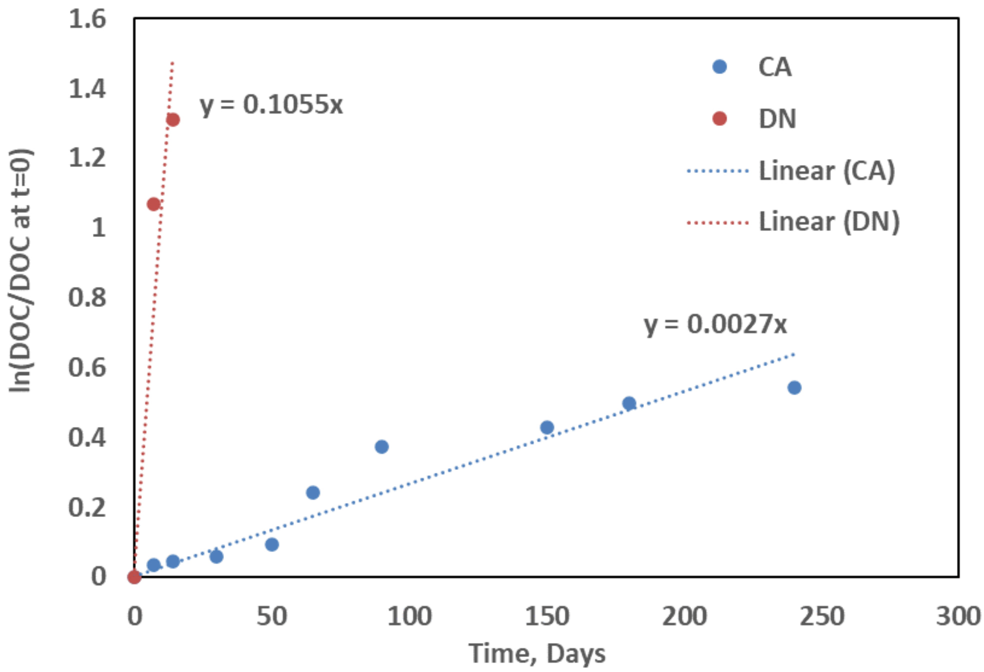

Figure S3. DOC degradation kinetics of 100 mg/L polyacrylic acid- (CA) and carboxylated dendrimeric- (DN) based antiscalants..

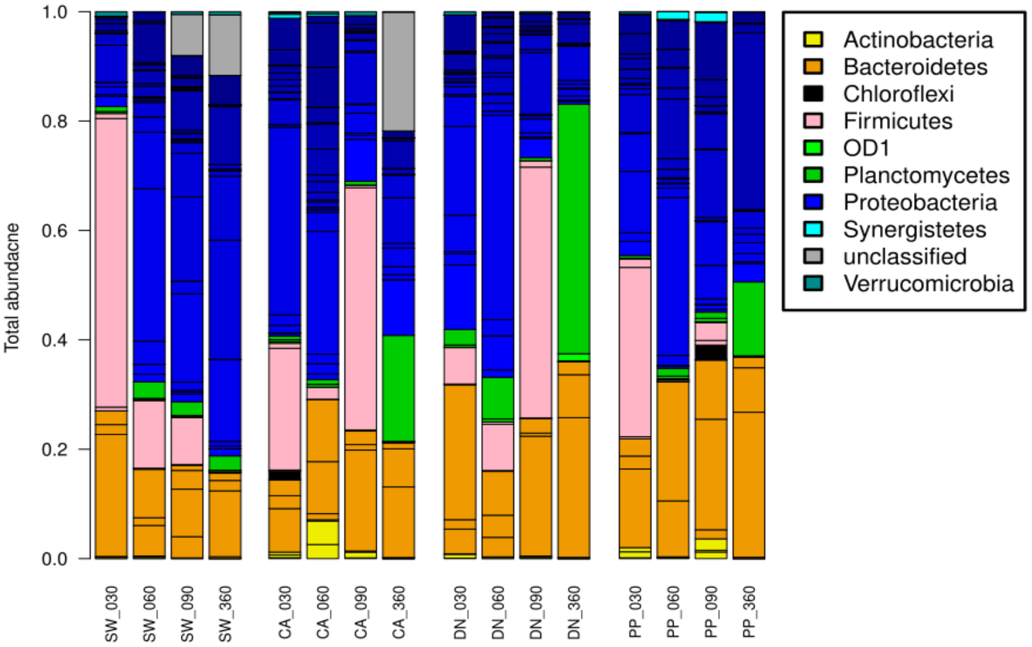

Figure S4. Phyla relative abundance in each treatment following different incubation periods. Shades of the same color represent the different bacterial order in each phylum.

Table S1. A number of high-quality sequences, Good's coverage, Chao1, and Shannon–Wiener Index for all studied treatment and different time points.

Days	Index	SW	CA	PP	Den
30 days	Sequences	6,628	10,184	11,187	21,150
	ESC	97.6	97.6	98.1	98.8
	Chao1	659	856	936	1065

	Shannon–Wiener	4.48	4.38	4.62	4.33
	Sequences	12,402	28,176	51,858	21,184
	ESC	98.1	98.6	98.9	98.7
60 days	Chao1	957	1,939	2,413	1,217
	Shannon–Wiener	4.00	4.00	3.47	3.68
	Sequences	8,337	8,332	33,267	7,117
	ESC	97.7	97.3	98.8	95.4
90 days	Chao1	814	1,704	1,722	829
	Shannon–Wiener	4.62	4.56	4.20	4.31
	Sequences	54,678	51,084	54,696	36,790
	ESC	98.8	98.9	98.7	98.1
360 days	Chao1	750	1,514	1,502	730
	Shannon–Wiener	4.72	4.21	4.31	4.55

Table S2. adonis significance based on Bray–Curtis distance matrix.

Df	SumsOfSqs	MeanSqs	F.Model	R2
	Pr(>F)			
map$antiscalant		3	0.4560	
0.15202		0.6855		0.11877
	0.754			
map$day			1	1.2287
	1.22870		5.5411	
0.31999		0.001 **		
map$antiscalant:map$day	3	0.3811		0.12704
	0.5729		0.09925	
0.934				
Residuals			8	1.7739
	0.22174			
0.46199				
Total				15
3.8398				
	1.00000			

Table S3. ANOVA analysis of Adjusted R2 for RDA analysis showing different variables and chemical analysis significance and percent of the variation.

DF	Variance	F		Pr(>F)	
Day		3	0.049184115	4.9018000	0.009 **
AS		3	0.012401975	0.9838548	0.429
DOC		1	0.007890386	1.8778446	0.160
TP		1	0.004889729	1.1637138	0.333
PO4		1	0.006653914	1.5835748	0.242
TDN		1	0.003962983	0.9431562	0.459
NH4		1	0.003121739	0.7429473	0.567
NO3		1	0.005428124	1.2918473	0.277
Residual	3	0.012605494	NA		NA
